# Multipopulational transcriptome analysis of post-weaned beef cattle at arrival further validates candidate biomarkers for predicting clinical bovine respiratory disease

**DOI:** 10.1038/s41598-021-03355-z

**Published:** 2021-12-13

**Authors:** Matthew A. Scott, Amelia R. Woolums, Cyprianna E. Swiderski, Andy D. Perkins, Bindu Nanduri, David R. Smith, Brandi B. Karisch, William B. Epperson, John R. Blanton

**Affiliations:** 1 Veterinary Education, Research, and Outreach Center, Texas A&M University and West Texas A&M University, Canyon, TX USA; 2 Department of Pathobiology and Population Medicine, Mississippi State University, Mississippi State, MS USA; 3 Department of Computer Science and Engineering, Mississippi State University, Mississippi State, MS USA; 4 Department of Comparative Biomedical Sciences, Mississippi State University, Mississippi State, MS USA; 5 Department of Animal and Dairy Sciences, Mississippi State University, Mississippi State, MS USA; 6 Department of Animal Sciences, Purdue University, West Lafayette, IN USA

**Keywords:** Transcriptomics, Infectious diseases, Biomarkers, Outcomes research, Inflammation, Innate immunity, Respiratory tract diseases, Respiratory signs and symptoms, Computational biology and bioinformatics

## Abstract

Bovine respiratory disease (BRD) remains the leading infectious disease in post-weaned beef cattle. The objective of this investigation was to contrast the at-arrival blood transcriptomes from cattle derived from two distinct populations that developed BRD in the 28 days following arrival versus cattle that did not. Forty-eight blood samples from two populations were selected for mRNA sequencing based on even distribution of development (n = 24) or lack of (n = 24) clinical BRD within 28 days following arrival; cattle which developed BRD were further stratified into BRD severity cohorts based on frequency of antimicrobial treatment: treated once (treated_1) or treated twice or more and/or died (treated_2+). Sequenced reads (~ 50 M/sample, 150 bp paired-end) were aligned to the ARS-UCD1.2 bovine genome assembly. One hundred and thirty-two unique differentially expressed genes (DEGs) were identified between groups stratified by disease severity (healthy, n = 24; treated_1, n = 13; treated_2+, n = 11) with edgeR (FDR ≤ 0.05). Differentially expressed genes in treated_1 relative to both healthy and treated_2+ were predicted to increase neutrophil activation, cellular cornification/keratinization, and antimicrobial peptide production. Differentially expressed genes in treated_2+ relative to both healthy and treated_1 were predicted to increase alternative complement activation, decrease leukocyte activity, and increase nitric oxide production. Receiver operating characteristic (ROC) curves generated from expression data for six DEGs identified in our current and previous studies (*MARCO, CFB, MCF2L, ALOX15, LOC100335828* (aka *CD200R1*)*,* and *SLC18A2*) demonstrated good-to-excellent (AUC: 0.800–0.899; ≥ 0.900) predictability for classifying disease occurrence and severity. This investigation identifies candidate biomarkers and functional mechanisms in at arrival blood that predicted development and severity of BRD.

## Introduction

Bovine respiratory disease (BRD) remains the leading cause of economic loss and antimicrobial usage in beef cattle production within North America^1–3^. Bovine respiratory disease is a multifactorial disease complex that results from a combination of host–pathogen interactions, host immunological and metabolic responses, and environmental conditions^4,5^. Diagnosis of BRD is most commonly made by visual identification of BRD-associated clinical signs which yields a diagnostic sensitivity of 27–62%^6,7^. The majority of clinical BRD cases occur within the first 3 weeks of facility arrival and visual assessment of clinical signs cannot diagnose subclinical BRD nor the long-term outcome of the disease^8–11^. Since the genomic mechanisms and immunologic events that confer resistance or susceptibility to clinical BRD remain disputed, analysis of the differences in whole blood transcriptomes assessed at facility arrival between cattle that develop BRD within 28 days of arrival and those that do not is likely to elucidate underlying mechanisms associated with BRD susceptibility and to inform the need for at arrival treatment.

Analyses of RNA sequencing (RNA-Seq) data have demonstrated the ability to provide sensitive and comprehensive interpretation of functional genomic mechanisms and molecular events occurring at a point in time^12^. RNA-Seq has been utilized in a number of experiments in an effort to identify host responses that are associated with clinical BRD. These studies have identified host immunological events following BRD-associated pathogen challenge^13–15^ as well as candidate biomarkers and genomic mechanisms from whole blood samples of post-weaned cattle^16–18^. However, RNA-Seq studies are most often performed with a subset of a single population that may not account for heterogeneity in gene expression across independent populations, and such investigations are generally underpowered^19–22^. Therefore, building upon our previous research^16,17^, we aimed to contrast the at-arrival whole blood transcriptomes of post-weaned beef cattle that developed BRD within the first 28 days of arrival and cattle that failed to develop BRD in this period from two independent populations.

The objectives of this study were to classify, discover, and further validate BRD-associated genes and mechanisms at facility arrival in post-weaned beef cattle in independent populations. Our approach was to profile and compare at-arrival whole blood transcriptomes of post-weaned beef cattle that failed to develop signs associated with clinical BRD and cattle that ultimately were diagnosed and treated for clinical BRD within the first 28 days of facility arrival. Additionally, cattle identified with clinical BRD were further stratified into BRD-associated severity cohorts, based on frequency of antimicrobial treatment, clinical evaluation scores, weight gain records, and mortality. This study identifies key differentially expressed genes and biological events which may influence BRD development and clinical severity. The findings from this study may serve as a foundation for advancing prognostic and therapeutic strategies against clinical BRD in post-weaned beef cattle.

## Materials and methods

### Animal use and study enrollment

All animal use and procedures were approved by the Mississippi State University Animal Care and Use Committee (IACUC protocol #17-120) and carried out in accordance with relevant IACUC and agency guidelines and regulations. This study was carried out in accordance with Animal Research: Reporting of In Vivo Experiments (ARRIVE) guidelines (https://arriveguidelines.org). This experiment was performed in conjunction with two additional studies examining the effect of at-arrival vaccination and deworming on health and performance outcomes (2017) and the impact of the density of cattle in pens on health and performance outcomes (2019). The 2017 study enrolled 80 commercial crossbred bulls (n = 57) and steers (n = 23), which were received over a course of 2 days. The 2019 study enrolled 199 commercial crossbred steers (one animal that was BVDV+ on ear notch antigen ELISA was removed), which were received over a course of 7 days. All animals in these studies were sourced from commercial livestock auctions and housed at the H. H. Leveck Animal Research Center at Mississippi State University (Starkville, MS, USA). On day 0 of each experiment, animals in both studies had blood samples collected into Tempus tubes (Applied Biosystems) via jugular venipuncture, which were frozen and stored at − 80 °C until analysis. On day 0, animals were vaccinated and administered oral anthelminthic, selectively in 2017, and to all animals in 2019. Bull calves were surgically castrated. Vaccinated animals received commercial vaccines subcutaneously at arrival and at 4 weeks post-arrival, per label instruction (Express 5, Boehringer Ingelheim Vetmedica; Vision 6 with SPUR, Merck Animal Health). Dewormed animals received oral fenbendazole and levamisole at arrival, at 5.0 mg/kg PO and 8.0 mg/kg PO, respectively. Fecal egg counts (FEC), rectal temperatures, and body weights were recorded for all animals at arrival. All cattle were given identification ear tags and confirmed to be negative for persistent infection with bovine viral diarrhea virus (BVDV) via ear notch antigen capture ELISA. Individual body weights were collected every 2 weeks in each study.

All animals were visually assessed each day for signs of BRD and/or other disease by trained university employees. The observed signs of BRD were assigned a severity score of 0–4, adapted from the scoring system described by Holland and colleagues^23^. Antimicrobial therapy was instituted according to severity score as described by Woolums and colleagues^24^. Briefly, all treated cattle in 2017 were given first treatment scores of 1 (Supplementary Table S1). Three of these cattle received two or more antimicrobial treatments for BRD (i.e., treated_2+). Four of the twelve cattle treated in 2019 were given first treatment scores greater than 1 (three received scores of 2 and one received a score of 3), and seven cattle required two or more antimicrobial treatments (Supplementary Table S2). Animals whose signs of BRD persisted following the final antimicrobial treatment were monitored daily for predetermined endpoints that are indicative of unlikely recovery. Such endpoints included severe dyspnea, moribund behavior, dull mentation, and/or signs of abnormal aggression. Animals deemed unlikely to recover were euthanized (four individuals; all from 2019) by project veterinarians via intravenous administration of pentobarbital followed by a gross necropsy that was performed by a board-certified veterinary anatomical pathologist.

### RNA sequencing and data processing

Twelve animals that remained healthy and twelve animals that developed BRD were selected for RNA-sequencing from each of the two cattle populations (2017 and 2019), yielding a total of 48 samples for sequencing. From the 2017 cohort of 80 cattle, the vaccination and deworming status could be aligned for 10 of the 12 animals in the healthy and BRD groups (Supplementary Table S1). Of the remaining two individuals in the 2017 healthy group, one was vaccinated and not dewormed, and the other was dewormed but not vaccinated. By contrast, the remaining two individuals in the 2017 diseased group were both vaccinated and dewormed. As all animals in the 2019 population were vaccinated and dewormed at arrival, the twelve healthy animals were selected randomly and the twelve BRD samples were selected based on varying frequencies of treatments (Supplementary Table S2). Additional information regarding weight gain, at-arrival fecal egg counts, rectal temperatures, and time of treatments and/or euthanasia for the cattle selected for RNA-Seq analysis is found in Supplementary Tables S1 and S2. RNA isolation, mRNA library preparation, and sequencing were performed at the UCLA Technology Center for Genomics and Bioinformatics (UCLA TCGB; Los Angeles, CA, USA). RNA was isolated from the Tempus blood tubes using the Tempus Spin RNA Isolation Kit (Applied Biosystems). RNA quantity and quality were measured using Agilent 2100 Bioanalyzer (Agilent); all RNA samples were of high quality (RIN: 8.3–9.5; mean = 8.9, s.d. = 0.3). Library construction was performed with the TruSeq RNA Sample Library Kit (Illumina), followed by 150 bp paired-end sequencing performed with an Illumina NovaSeq 6000 (v1.7; S4 reagent kit v1.5) in one lane.

Quality assessment of raw and trimmed sequenced reads was performed with FastQC v0.11.9^25^. Quality filtering and adapter trimming of raw sequenced reads was performed with Trimmomatic v0.39^26^. Briefly, trimming was performed by scanning each read with a 4-base pair sliding window and removing read segments below a minimum base quality score of 20 and a read length of less than 32 bases. Trimmed reads were mapped to the bovine reference assembly ARS-UCD1.2 using HISAT2 v2.2.1^27,28^. Mapping alignment statistics indicated an average overall alignment rate exceeding 97% (Supplementary Table S3). Transcript/gene assembly and quantification was performed with StringTie v2.1.2^29,30^; a gene-level raw count matrix was generated for each sample with the program prepDE.py^31^.

### Differential gene expression analysis

Raw gene counts generated for each sample were processed and analyzed in R v4.0.2 with the Bioconductor package edgeR v.3.30.3^32,33^. Samples were placed into three different cohorts based on BRD status, frequency of treatments, and mortality. Briefly, cattle never diagnosed with clinical BRD nor treated with antimicrobials were categorized as “healthy” (n = 24), those diagnosed with clinical BRD and only treated with antimicrobials one time were categorized as “treated_1” (n = 13), and those diagnosed with BRD, treated with antimicrobials more than once, and/or euthanized due to worsening BRD state were categorized as “treated_2+” (n = 11); four individuals from 2019 (IDs: 155_2019, 175_2019, 252_2019, and 274_2019) were those cattle which were euthanized. All animals classified with clinical BRD were diagnosed and treated within the first 14 days post-arrival. Raw counts were processed and filtered according to procedures described by Chen and colleagues^34^, utilizing gene counts-per-million (CPM) of 0.5 across a minimum of three samples; any gene not meeting these criteria was excluded from analysis. Library normalization for edgeR analysis was performed with the trimmed mean of M-values method (TMM)^35^. Differentially expressed genes (DEGs) were identified through pairwise comparison of the three groups using likelihood ratio testing (glmLRT); DEGs from edgeR analysis were considered significant with a false discovery rate (FDR) of ≤ 0.05^36^. The processed and filtered raw gene counts were subsequently utilized to test for further validation of DEGs, incorporating potential confounding variables reported from our principal component analysis (see methods below). The Bioconductor package DESeq2 v1.30.0^37^ was employed to identify DEGs while accounting for castration status at arrival (Sex), at-arrival parasite egg counts per gram of feces (FEC), and population (Year) in a reduced formula. Specifically, we evaluated for differences in gene expression between the three BRD severity cohorts with negative binomial generalized linear model (GLM) likelihood ratio testing, implementing a multifactorial analysis of deviance approach (ANODEV)^37,38^. Model fitting, count normalization, and dispersion estimation was performed with default procedures as detailed by Love and colleagues^37^. DEGs from DESeq2 multifactorial analysis were considered significant with an FDR ≤ 0.10.

### Data characterization: heatmap, principal component, and receiver operating characteristic curve analyses

Visual comparisons of the differentially expressed genes between all three groups was performed with the Bioconductor package VennDiagram v1.6.20^39^. Heatmaps of DEGs were generated with the Bioconductor package pheatmap v1.0.12^40^, utilizing Minkowski distances and Pearson correlation coefficients for unsupervised hierarchical clustering of samples and DEGs, respectively. Further heatmap visualization and clustering was performed with all filtered and normalized gene counts (16,346), empirically grouping gene expression into 12 distinct clusters with the k-means algorithm embedded within pheatmap; Minkowski distances and Pearson correlation coefficients were utilized for unsupervised hierarchical clustering of samples and gene clusters, respectively. Data exploration and dimensional reduction via principal component analysis (PCA) was conducted with the Bioconductor package PCAtools v2.0.0 using the correlation matrix^41^. Gene counts were normalized through mean centering and variance scaling. Metadata components from all cattle for PCA correlation analysis included at-arrival parasite egg counts per gram of feces (FEC), castration status at arrival (Sex), average daily weight gain in pounds (ADG), time to first treatment (Timing), population (Year), and disease severity (Group). The bottom 10% of genes, those with the lowest variance, were removed prior to analysis. All information regarding these components is available in Supplementary Tables S1 and S2.

Genes counts from this study (log2 normalized) were obtained from DEGs which overlapped, in terms of expression (fold change direction) and significance (FDR ≤ 0.05), in this and our previous study^16^. All 132 DEGs were analyzed for classification capability, but six genes were given selection priority based on overlap this and our previous study^16^: *MARCO, CFB, MCF2L, ALOX15, LOC100335828* (*CD200R1*)*,* and *SLC18A2*. To evaluate these DEGs as potential biomarkers that predict likelihood to develop BRD, and to differentiate BRD severity outcomes, multiclass receiver operating characteristic (ROC) curve analysis of DEGs was performed with the Bioconductor package pROC v1.16.2^42^. Aggregate area under the curve (AUC) was calculated for each ROC curve. An ROC curve cross-validation analysis was performed to evaluate predictive capability across each population (year) independently; ROC curve analysis from one population was utilized to generate log2CPM cutoffs, which were applied to the other population for classificational assessment. Sensitivity, specificity, positive/negative predictive value, and balanced accuracy [(sensitivity + specificity)/2] was calculated for cross-validation. The ability to discriminate between the three groups (healthy, treated_1, and treated_2+) was evaluated as “excellent” (AUC: ≥ 0.900), “good” (AUC: 0.899–0.800), “fair” (AUC: 0.799–0.700), “poor” (AUC: 0.699–0.600), or “failed” (AUC: < 0.600)^43^. Color scaling for all packages was performed with the Bioconductor package viridis v0.5.1^44^ to allow ease of visual interpretation for individuals with color blindness.

### Enrichment, network, and regulatory analyses of DEGs

Gene Ontology (GO) enrichment analysis was performed with WebGestalt 2019 (WEB-based GEne SeT AnaLysis Toolkit; accessed January 26, 2021), utilizing human orthologs and their functional databases^45^. The DEGs identified from edgeR in each differential gene expression analysis (treated_1 versus healthy, n = 64; treated_2+ versus healthy, n = 40; treated_2+ vs treated_1, n = 81; Supplementary Table S4) were utilized for GO terms enrichment for each comparison. Overrepresentation analysis parameters within WebGestalt 2019 included between 5 and 3000 genes per category (Benjamini–Hochberg procedure for multiple hypothesis correction, and FDR cutoff of 0.05 for significance). Pathway enrichment analysis performed within WebGestalt 2019 utilized the pathway database Reactome^46^. The DEGs found between each comparison were utilized for pathway enrichment, similar to GO term enrichment analysis. Enriched GO terms and pathways were evaluated for directionality (increased or decreased) based on log2 fold changes of associated DEGs.

Protein–protein interactions of DEGs were evaluated with the Search Tool for the Retrieval of Interacting Genes (STRING) database v11.0^47^, employing human orthologs of all 132 uniquely identified DEGs. Briefly, interactions were considered significant with a combined interaction score of 0.400 (medium confidence) and network clustering was performed with the k-means algorithm within the STRING interface empirically pre-set to six clusters; disconnected nodes were omitted from the STRING interaction network. Directionality (increased or decreased) of proteins within each cluster was based on log2 fold change of associated DEGs within each cohort comparison. Functional association was made from the STRING cluster enrichment tables.

Ingenuity Pathways Analysis (IPA; Qiagen) was used for upstream regulator and function process analyses of DEGs, utilizing the Benjamini–Hochberg method for multiple hypothesis correction and calculated z-score cutoffs of ± 2.0.

## Results

### edgeR differential gene expression analysis and data visualization

**Figure 1:**
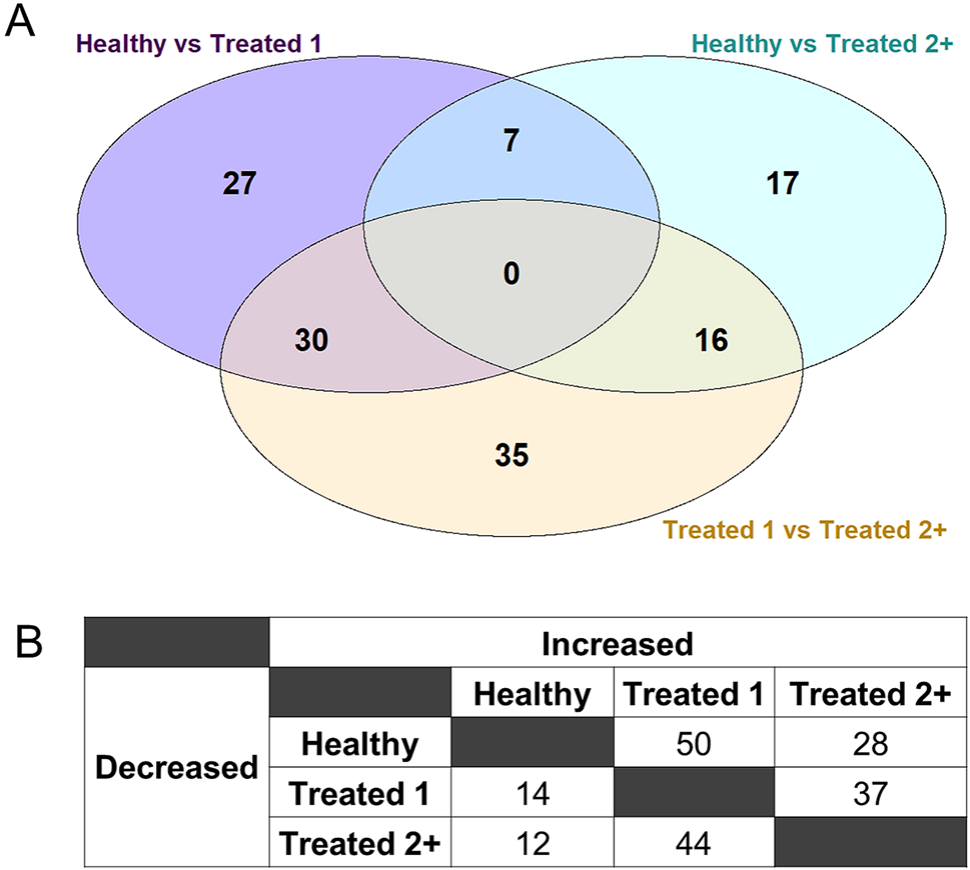
Number of DEGs (FDR ≤ 0.05) identified at arrival through pairwise comparisons of all three disease severity cohorts. (**A**) Venn diagram representing the number of DEGs shared and distinct to each comparative analysis. (**B**) Relative directionality (log2 fold change) of DEGs identified in each severity cohort.

**Figure 2:**
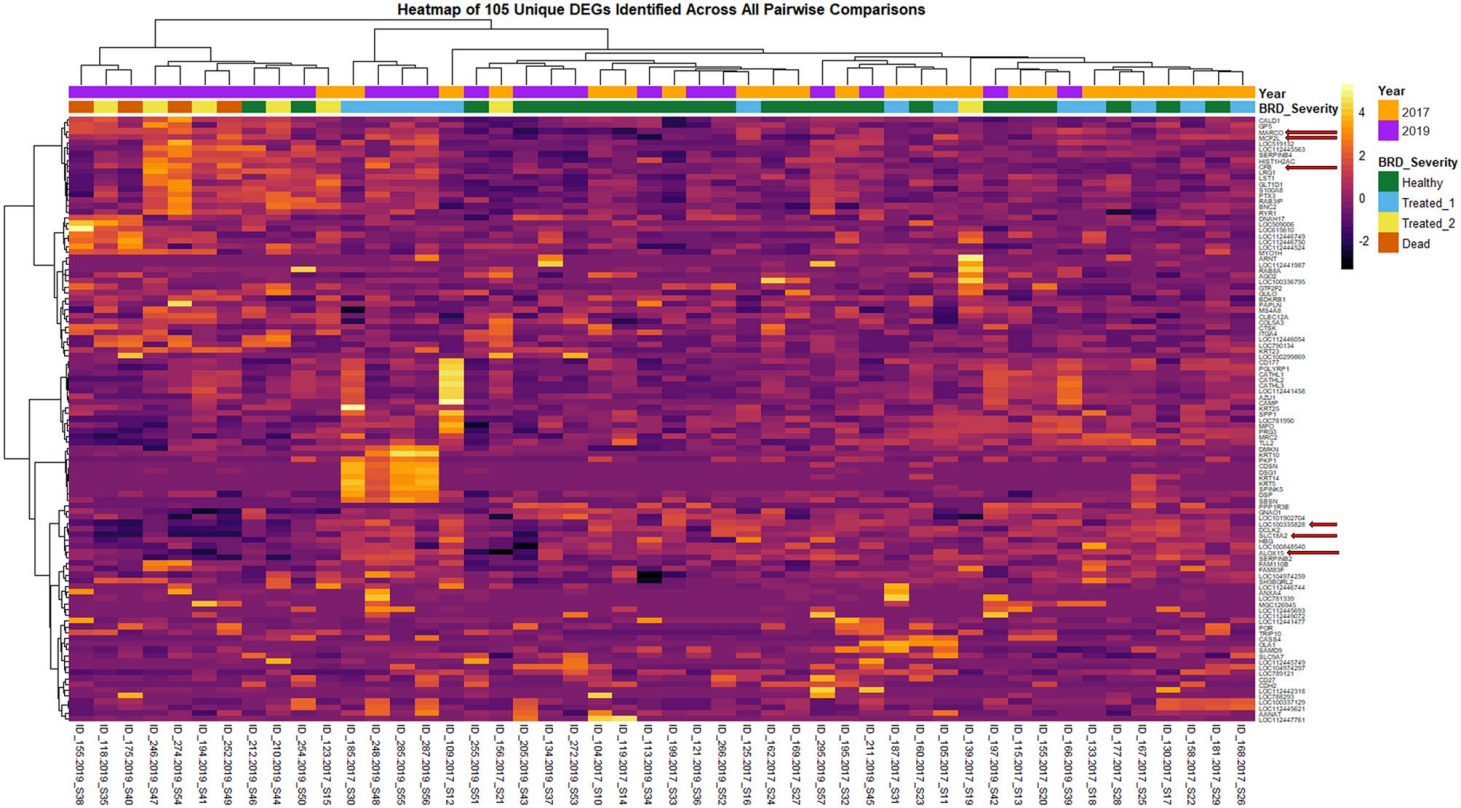
Heatmap and hierarchical clustering analyses of gene expression profiles across all 48 sample libraries with 105 unique DEGs identified. Heatmapping of the DEGs was performed with the z-scores calculated from Trimmed Mean of M-values (TMM) normalized counts. Samples were labeled with population (year) and severity (including mortality) to illustrate differences in expression patterns. Yellow/white: relative high expression; purple/black: relative low expression. Arrows indicate genes chosen for ROC curve analysis, based on overlapping findings from our previous research^16^.

Alignment of reads to the ARS-UCD1.2 bovine genome assembly and gene count matrix generation yielded 28,478 unique annotated genes. Following pre-processing of genes with low expression across samples, a total of 16,346 annotated genes were analyzed for differential gene expression; the 48 samples possessed a total of 209.4 million gene counts, with a median library size of 43.6 million gene counts (Supplementary Fig. S1). In total, edgeR analysis identified 132 unique genes (FDR ≤ 0.05) as differentially expressed in at least one of the 3 comparisons of treated_1, treated_2+, and healthy groups, as indicated in Supplementary Table S4. Sixty-four DEGs were identified between treated_1 versus healthy, 40 DEGs between treated_2+ versus healthy, and 81 DEGs between treated_2+ versus treated_1 (Fig. 1A,B). No DEGs were common to all comparisons. Figure 2 is a heatmap of normalized expression data from all cattle for 105 genes that were differentially expressed across the comparisons with the most severely diseased cattle (treated_2+ vs healthy, treated_2+ vs treated_1). Hierarchical clustering of the gene expression patterns depicts healthy and treated_1 cattle as the most similar at the right of the map, while treated_2+ cattle and most severely diseased cattle (i.e., euthanized), tended to cluster distinctly to the left of the map (Fig. 2). Similarly, this distinction of cattle which develop the most severe BRD is apparent, although less prominent, with hierarchical clustering of samples from normalized counts of all 16,346 genes (Supplementary Fig. S2).

### Principle component analysis

**Figure 3:**
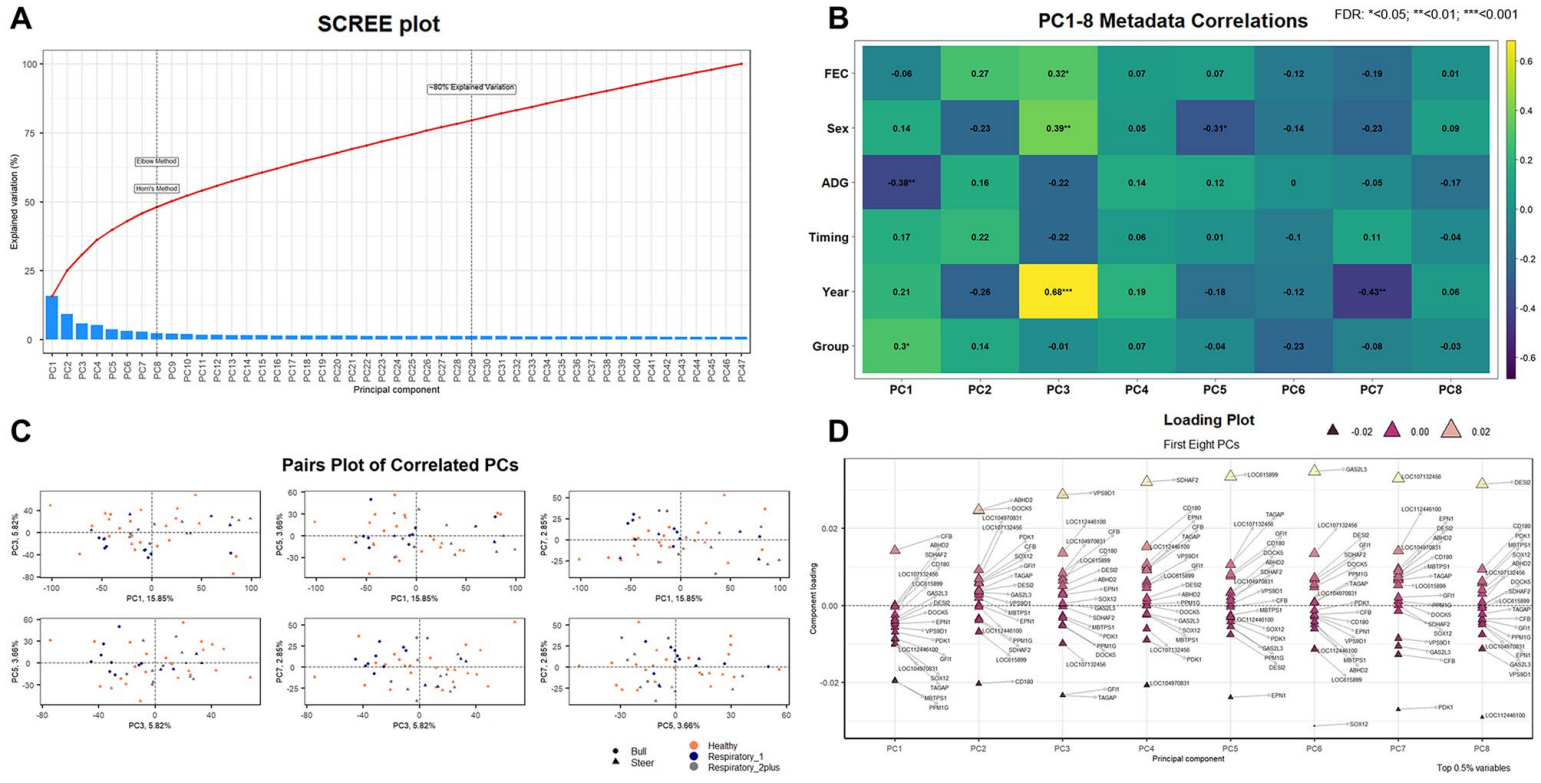
Principal component analysis of gene expression data generated for each sample. (**A**) Scree plot showing all components which account for the total variance within the data set. The Elbow and Horn’s parallel analysis methods were used to determine the optimum number of components to retain. The first eight PCs retained accounted for ~ 48% of the total variance in the data set, while 80% of explained variation would have required 29 PCs. (**B**) Exploration of Pearson correlation coefficients associated with metadata components for the first eight PCs. Each animal’s fecal egg count at arrival, castration status at-arrival, average daily gains for the first 14 days, population (year), and relative disease severity over the course of the study were clinical aspects that possessed significant association with one or more PCs. Time to first treatment was not significantly correlated with any of the first eight PCs. (**C**) Pairs plot (multi-biplot) graph of PCs possessing significant metadata correlations. Each dot (vector) represents the PC score of an individual sample, where the further away the vector is from the PC center, the more influence that vector possesses for the PC. (**D**) Loading plot with annotated variables (genes) driving variation in the first eight PCs. These plots are used to identify the top 0.5% of genes found to be the most responsible for driving variation within each PC.

Metadata from cattle for the variables at-arrival parasite egg counts per gram of feces (FEC), castration status at arrival (Sex), average daily weight gain in pounds (ADG), time to first treatment (Timing), population (Year), and disease severity (Group) can be found in Supplementary Tables S1 and S2. Using the combination of the Elbow method and Horn’s parallel analysis^48,49^, the first eight principal components, which accounted for 48% of the variance in the data, were chosen (Fig. 3A). The first principal component (PC) explained 16% of the data variance and was positively correlated with Group (*r* = 0.30, FDR < 0.05) and negatively correlated with ADG (*r* = − 0.38, FDR < 0.01) (Fig. 3A,B). The PC3, which accounted for 6% of the variance, included the strongest and most significant correlation observed, which was with Year (*r* = 0.68, FDR < 0.001), and less significant correlations with Sex (*r* = 0.39, FDR < 0.01) and FEC (*r* = 0.32, FDR < 0.05). Two PCs, PC5 and PC7, were negatively correlated with Sex (*r* = − 0.31, FDR < 0.05) and Year (*r* = − 0.43, FDR < 0.01), respectively (Fig. 3B). Pair plots of the four PCs with significant correlations with metadata components (PCs 1, 3, 5, and 7) demonstrated no discernable patterns across the 48 cattle, when accounting for Group and Sex (Fig. 3C).

Genes providing the greatest contributions to each PC are indicated in the loading plots (Fig. 3D). Genes with the greatest influence on PC1, the only PC significantly correlated to measures of disease severity, include *PPM1G, MBTPS1, SOX12, TAGAP*, and *CFB*. Genes with major influence on PC3, which correlated to Year, FEC and Sex, included *TAGAP, GF11, CFB*, and *VPS9D1*. *EPN1* and *TAGAP* had the greatest influence on PC5, which was significantly correlated with Sex. *PDK1, CFB*, *GAS2L3, DESI2* and *EPN1* accounted for the greatest variance in PC7 which correlated with Year.

### Gene selection and receiver operating characteristic (ROC) curve analyses

**Figure 4:**
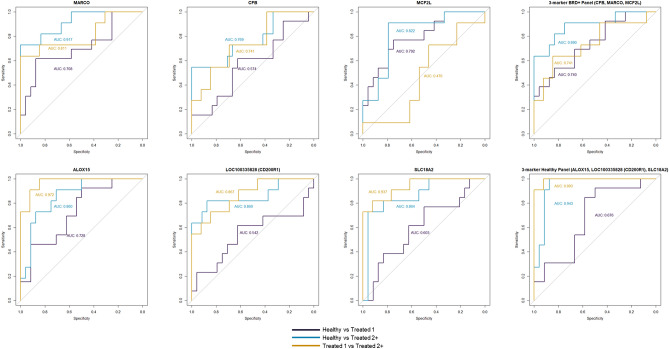
Receiver operator characteristic (ROC) curves with calculated area under the curve (AUC) of selected DEGs. Hypothetical three-marker panels were generated based on expression trends across disease severity cohorts. *MARCO, CFB,* and *MCF2L* were DEGs relatively increased in gene expression as BRD severity increased. *ALOX15, LOC100335828,* and *SLC18A2* were DEGs relatively decreased in gene expression as BRD severity increased.

To evaluate DEGs as potential biomarkers that predict individuals that are likely to develop BRD, and to differentiate BRD severity outcomes, multiclass ROC curves were generated with expression data from the 48 individual cattle (Fig. 4); complete ROC curve analysis across all 132 DEGs is found in Supplementary Table S5. Based upon differential expression patterns in this investigation and in our previous work^16^, six genes were of particular interest: *ALOX15, MARCO*, *CFB*, *MCF2L*, *LOC100335828* (*CD200R1*)*,* and *SLC18A2*. In discriminating cattle that would become severely diseased (treated_2+) from those that remain healthy, *MARCO* expression demonstrated excellent discrimination (AUC: 0.917), followed by expression of *SLC18A2* (AUC: 0.864), *ALOX15* (AUC: 0.860), *LOC100335828* (AUC: 0.860) and *MCF2L* (AUC: 0.822), which independently provided good discrimination. *CFB* expression provided fair discrimination for these two groups (AUC: 0.769). A combination of *ALOX15*, *LOC100335828*, and *SLC18A2* expression (“3-marker Healthy Panel”) provided excellent discrimination of cattle that would become severely diseased (treated_2+) from those that would remain healthy (AUC 0.943). The combination of *MARCO*, *CFB*, and *MCF2L* (“3-marker BRD + Panel”) also provided good discrimination between these two groups (AUC: 0.890).

In predicting cattle that became more severely diseased (treated_2+) from those that developed less severe disease (treated_1), *ALOX15* (AUC: 0.972) and *SLC18A2* (AUC: 0.937) expression independently provided excellent discrimination. As independent predictors, discrimination of these two groups by *LOC100335828* (AUC: 0.867) and *MARCO* (AUC: 0.811) expression was good while discrimination based on *CFB* (AUC: 0.741) expression was deemed fair. The combination of *ALOX15*, *LOC100335828*, and *SCL18A2* expression provided excellent discrimination in predicting treated_2+ versus treated_1 cattle (AUC 0.993), while the combination of *MARCO*, *CFB*, and *MCF2L* expression provided fair discrimination of these two groups (AUC: 0.741).

For differentiating cattle that would become less severely diseased (treated_1) from cattle that would remain healthy, *MCF2L* expression was the best independent discriminator (AUC: 0.792) followed by *ALOX15* (AUC: 0.728), and *MARCO* (AUC: 0.708). Of the two multi-gene panels, only the 3 Marker BRD + Panel of *ALOX15*, *LOC100335828*, and *SLC18A2* demonstrated acceptable discrimination (AUC: 0.740).

Single-population ROC curve analyses of these six genes were performed to independently determine BRD cohort classificational ability of these six genes and generate log2CPM cutoffs for cross-validation (Supplementary Table S6). *ALOX15* provided excellent discrimination of treated_2+ cattle compared to both healthy and treated_1 cattle in 2017 and treated_1 cattle in 2019. Independently, the combination of *ALOX15*, *LOC100335828*, and *SCL18A2* (“Healthy” panel) provided good-to-excellent discrimination of treated_2+ cattle compared to both healthy and treated_1 cattle in 2017 and 2019. The three genes comparatively upregulated in diseased cattle (*MARCO*, *CFB*, and *MCF2L*) better discriminated severity cohorts in 2019 compared to 2017. Generally, the 2019 population better discriminated severity cohorts compared to healthy cattle (AUC: ≥ 0.800), when contrasted to the 2017 population.

The AUC cutoffs (log2CPM) generated from the single-population ROC curve analyses was applied to the contrasting population for cross-validation of these six genes (Supplementary Table S7). *MARCO*, *CFB*, and *MCF2L*, DEGs comparatively increased in diseased cattle, and better classified treated_2+ cattle compared to healthy in the 2019 population versus the 2017 population. Additionally, these three genes often failed to classify treated_2+ compared to treated_1 cattle in the 2017 population (sensitivity: 0.000; balanced accuracy: ≤ 0.500). Similar to the single-population ROC curve analyses, *ALOX15* and the “Healthy” panel performed well in classifying treated_2+ cattle from both healthy and treated_1 cattle.

### Gene ontology and pathway enrichment analyses

Analysis of GO terms from DEGs identified between treated_1 and healthy cattle identified 50 biological process terms, 25 cellular component terms, and 4 molecular function terms that were significantly over-represented (Supplementary Table S8). Biological processes identified from DEGs between these two groups were related to epidermal cornification and keratinization, neutrophil activation and degranulation, humoral immune response, and host defense against microorganisms (e.g., bacteria). Cellular components identified from DEGs between these two groups involved exosomes, secretory granules, lysosomes, and intracellular vesicles. Molecular functions identified from DEGs between these two groups involved glycosaminoglycan/lipopolysaccharide binding, heparin binding, and the structural constituent of cytoskeleton. These GO terms were enriched by DEGs comparatively increased in treated_1 cattle. No significantly enriched GO terms were identified from DEGs derived from the treated_2+ versus healthy comparison (FDR < 0.05; Supplementary Table S9). Analysis of GO terms from DEGs identified between treated_2+ and treated_1 cattle identified 41 biological process terms, 33 cellular component terms, and 1 molecular function term as significantly over-represented (Supplementary Table S10). Enriched biological processes identified from DEGs between these two groups were related to epidermal cornification and keratinization, neutrophil activation and degranulation, antimicrobial humoral immunity, and granulocyte activation. Cellular components identified from DEGs between these two groups involved secretory granules, cornified envelopes, supermolecular complexes, ficolin-1 granules, and cytoskeletal components. A single significant molecular function, inhibition of serine-type endopeptidases, was identified from the DEGs between these two groups. These GO terms were enriched by DEGs comparatively decreased in treated_2+ cattle.

Five significantly enriched pathways were identified from the DEGs between treated_1 versus healthy cattle (Supplementary Table S8). The enriched pathways identified from DEGs between these two groups involved the formation of cornified envelopes, neutrophil degranulation, keratinization, apoptotic cleavage of cell adhesion proteins, and innate immunity, all of which were driven by DEGs comparatively increased in treated_1. No pathways were significantly enriched from DEGs identified between treated_2+ and healthy cattle (Supplementary Table S9). Six significantly enriched pathways were identified from the DEGs between treated_2+ versus treated_1 cattle (Supplementary Table S10). The enriched pathways identified from DEGs between these two groups involved neutrophil degranulation, the formation of cornified envelopes, keratinization, innate immunity, and apoptotic cleavage of cell adhesion proteins, all of which were comparatively decreased in treated_2+.

### Protein–protein interaction network analysis

**Figure 5:**
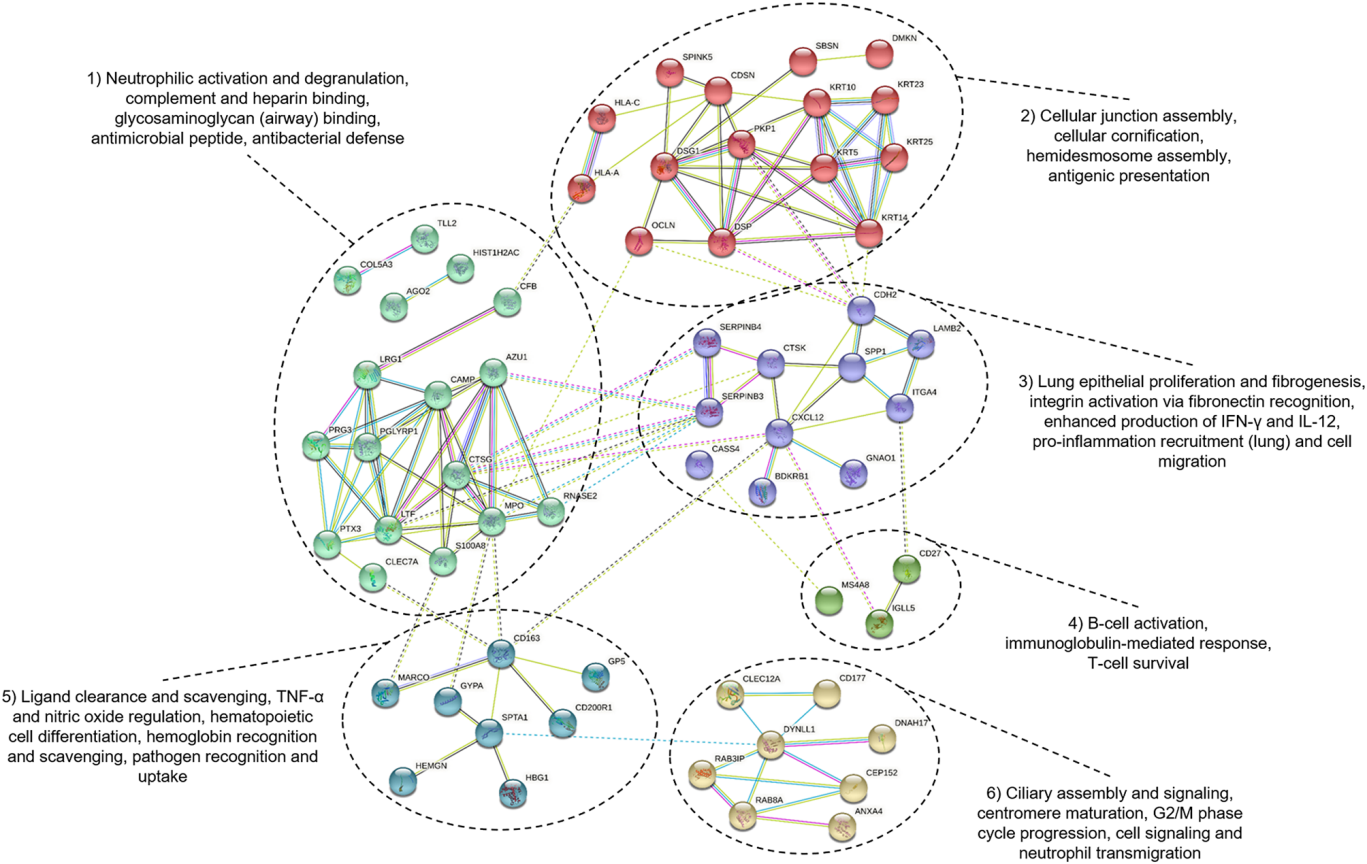
Protein–protein interaction network generated from DEGs. K-means clustering was performed based on product functionality. Product function of each cluster is based on nodal (gene product) information from curated literature and database mining in STRING. The color and number of edges (lines) corresponds with the type and strength of data supporting an interaction between two nodes. Known interactions are colored teal (curated database) and purple (experimental evidence). Predicted interactions are colored green (gene neighboring), red (gene fusion), and dark blue (gene co-occurrence). All other interaction evidence is colored yellow/lime green (text mining), black (co-expression association), and light blue (protein homology). Filled nodes represents that a three-dimensional structure is known or predicted.

All 132 unique DEGs were included for protein–protein interaction networking. Unique DEGs mapped to 102 homologous gene products (nodes) with 130 generated associations (edges) within STRING. When disconnected nodes were removed, the interaction network included 62 nodes with known and predicted protein interactions. All gene product identifiers (nodes) and interaction scores are presented in Supplementary Table S11. The resulting matrix was clustered into six distinct functional groups, presented in Fig. 5. The teal cluster (#1), driven primarily by DEGs increased in treated_1 relative to both treated_2+ and healthy cattle, is functionally associated with neutrophil enhancement and innate antimicrobial defense. The red cluster (#2), driven primarily by DEGs increased in treated_1 relative to both treated_2+ and healthy cattle, is functionally associated with cellular adhesion, cornification, and antigenic presentation. The purple cluster (#3) is associated with pro-inflammatory cytokine signaling and extracellular matrix protein production and binding (fibronectin, integrin) in lung tissue. The green cluster (#4) is functionally associated with B-cell activation and T-cell survivability. The blue cluster (#5), driven by DEGs increased in treated_2+ relative to both treated_1 and healthy cattle, is associated with increased apoptotic cell/ligand clearance, macrophage activity (via pathogen recognition/uptake and hemoglobin recognition), and regulation of TNF-alpha and nitric oxide. The yellow cluster (#6) is functionally associated with cell interphase and neutrophilic transmigration.

### Ingenuity pathway analysis

Ingenuity Pathway Analysis (IPA) was further utilized to identify enriched functional classifications from DEGs identified in each disease cohort comparison, shown in Table 1. Modeling of DEGs derived from treated_1 versus healthy cattle indicated increased activities that would attract leukocytes, including the recruitment of neutrophils. Modeling of DEGs from treated_2+ versus treated_1 cattle indicated that leukocyte recruitment and activation, including the recruitment of neutrophils, was decreased, while nitric oxide production was increased. No significant functional annotations were identified from modeling the DEGs from treated_2+ versus healthy cattle.

**Table 1:** Functional annotation analysis from DEGs identified between cohorts using Ingenuity Pathway Analysis (IPA). Annotations were considered significant with an adjusted *p*-value ≤ 0.05 and an activation z-score of ≥ 2 or ≤ − 2.

Diseases or functions annotation	Cohort	Predicted activation state	Activation z-score	*p*-value
Leukocyte migration	Treated 1 (vs Healthy)	Increased	2.780	1.74E−03
Cell movement of leukocytes	Treated 1 (vs Healthy)	Increased	2.774	7.78E−04
Cell movement of phagocytes	Treated 1 (vs Healthy)	Increased	2.433	2.70E−03
Cell movement of neutrophils	Treated 1 (vs Healthy)	Increased	2.417	1.05E−04
Cellular infiltration by leukocytes	Treated 1 (vs Healthy)	Increased	2.410	7.54E−04
Migration of cells	Treated 1 (vs Healthy)	Increased	2.404	2.80E−03
Cell movement of mononuclear leukocytes	Treated 1 (vs Healthy)	Increased	2.377	9.24E−04
Cell movement	Treated 1 (vs Healthy)	Increased	2.298	6.59E−03
Recruitment of neutrophils	Treated 1 (vs Healthy)	Increased	2.190	3.06E−05
Cell movement of T lymphocytes	Treated 1 (vs Healthy)	Increased	2.175	2.25E−04
Cell movement of monocytes	Treated 1 (vs Healthy)	Increased	2.166	1.54E−05
Chemotaxis of mononuclear leukocytes	Treated 1 (vs Healthy)	Increased	2.162	2.38E−05
Chemotaxis of neutrophils	Treated 1 (vs Healthy)	Increased	2.156	1.59E−05
Cell movement of lymphocytes	Treated 1 (vs Healthy)	Increased	2.156	2.83E−03
Inflammatory response	Treated 1 (vs Healthy)	Increased	2.073	1.93E−03
Recruitment of leukocytes	Treated 2+ (vs Treated 1)	Decreased	− 2.395	2.32E−05
Recruitment of neutrophils	Treated 2+ (vs Treated 1)	Decreased	− 2.392	6.59E−06
Activation of leukocytes	Treated 2+ (vs Treated 1)	Decreased	− 2.049	1.41E−03
Synthesis of nitric oxide	Treated 2+ (vs Treated 1)	Increased	2.176	4.08E−04

Further employment of IPA classified upstream regulators based on the DEGs identified in each disease cohort comparison. The significant upstream regulators that may explain observed gene expression changes (Z-score > 2 or < − 2, *p*-value < 0.05) are presented in Table 2. The transcription regulators *MRTFB, SRF,* and *MRTFA* were predicted to be inhibited in treated_1 cattle when compared to healthy, resulting in the upregulation of genes associated with neutrophil activity and antimicrobial defense. *IL1A* and *FADD* were both predicted as activated upstream regulators in treated_2+ cattle when compared to treated_1, targeting genes primarily involved in inflammatory signaling and calcium binding.

**Table 2:** Significantly enriched upstream regulators among DEGs identified in disease severity cohorts using Ingenuity Pathway Analysis (IPA). Upstream regulators were considered significant with an adjusted *p*-value ≤ 0.05 and an activation z-score of ≥ 2 or ≤ − 2.

Upstream regulator	Cohort	Molecule type	Predicted activation state	Activation z-score	*p*-value of overlap	Target molecules
MRTFB	Treated 1 (vs Healthy)	Transcription regulator	Inhibited	− 2	1.73E−04	CAMP, CTSG, LTF, PGLYRP1
SRF	Treated 1 (vs Healthy)	Transcription regulator	Inhibited	− 2	4.61E−04	CAMP, CTSG, LTF, PGLYRP1
MRTFA	Treated 1 (vs Healthy)	Transcription regulator	Inhibited	− 2	9.96E−05	CAMP, CTSG, LTF, PGLYRP1
IL1A	Treated 2+ (vs Treated 1)	Cytokine	Activated	2	1.17E−04	CTSK, PTX3, S100A8, SERPINB4
FADD	Treated 2+ (vs Treated 1)	Other	Activated	2	7.87E−04	CTSK, KRT14, PTX3, SPP1

### DESeq2 differential expression analysis for DEG substantiation

To substantiate significant differential expression patterns seen between these BRD cohorts, we implemented DESeq2 for multifactorial ANODEV testing accounting for Sex, Year, and FEC as potentially confounding variables. This analysis yielded 93 unique genes (FDR ≤ 0.10) as differentially expressed between the three cohorts (Supplementary Table S12). When compared to the results from edgeR analysis, 66 genes were found to be overlapped in DESeq2 analysis and of equivocal expressional directionality. Notably, five of the six genes of particular interest were identified as differentially expressed in both edgeR and DESeq2 analyses (*ALOX15, MARCO*, *CFB*, *MCF2L*, and *SLC18A2*). Furthermore, genes involved in epidermal cornification and keratinization (*KRT5/10/14/25*, *CDSN*, *DSG1*, *DSP*, *SPINK5*), neutrophil activation and degranulation (*AZU1*, *CD177*, *MPO*, *PGLYRP1*, *PRG3*), and antimicrobial peptide production (*CAMP*, *CATHL2*/*3*) were found to be differentially expressed in both analyses.

## Discussion

In recent years, bovine RNA-Seq data have been utilized to identify biomarkers that are predictive of or associated with BRD. Previous studies have utilized tissue samples, such as lung, lymph node, and tonsil, at the time of peak clinical infection following challenge with individual BRD pathogens^13–15^. These studies provided important foundational efforts for identifying regulatory networks and host–pathogen interactions related to immunological defense at the time of peak clinical BRD. However, pathogen challenge models are limited in the degree to which they represent naturally occurring BRD, because they do not recapitulate the complex multifactorial nature of the disease^50–52^. In contrast, gene expression patterns have been analyzed in arrival blood from post-weaned beef cattle that later developed BRD^16–18^. These investigations of naturally occurring BRD assure the biological context that is necessary for gene expression analyses to identify altered biological systems for clinical BRD prediction. By testing large populations of BRD susceptible cattle, this approach allows differentiation of pervasive changes in biological systems that segregate with BRD prediction and staging, from other dynamic environmental and host factors that also alter biological systems but do not segregate with BRD. Using this approach, altered immunological and metabolic pathways, including those important to host–pathogen interactions, are recognized by their shared patterns in cattle that develop BRD. Patterns that influence clinical BRD outcomes and support the discovery of novel pathophysiology, candidate biomarkers and therapeutic modalities can also be realized^53,54^.

The identification of differentially expressed genes and enriched genomic mechanisms within whole blood samples supports the discovery and use of candidate biomarkers and novel therapeutic modalities. Whole blood is an easily obtainable, non-invasive sample type that represents transient biological occurrences at distinct physiological sites^55,56^. By analyzing transcriptomes in blood collected at arrival from two distinct populations of cattle, this investigation (1) corroborated our prior findings that at-arrival expression profiles of *MARCO, CFB, MCF2L, ALOX15, LOC100335828* (*CD200R1*)*,* and *SLC18A2* were common to cattle from two distinct populations that went on to develop BRD, (2) demonstrated using ROC curves that the at-arrival expression of these genes have predictive potential in classifying BRD acquisition and severity, and (3) derived novel genomic information relevant to BRD severity, related to leukocyte activity and airway epithelium.

A limitation of this study is the many factors that varied between individual cattle and between the populations which contribute to biological variation in the transcriptomes of individual cattle. In this regard, our principal component analysis identified population (Year) in PC3 as a significant and identifiable source of variation in gene expression (Fig. 3B). Our populations lacked information regarding treatment/vaccination of individual animals prior to arrival. Additionally, all individuals in this study were commercial cattle (e.g., unknown genetic characteristics), were likely to be at differing time points in the disease spectrum of BRD, and were likely to have a host of other factors that varied between individuals prior to their arrival. Relevant to this variation is our overarching objective, which is to identify arrival gene expression that predicts BRD outcome in commercial beef cattle within 28 days following arrival. To achieve this objective, it is essential that the very factors causing variation in the transcriptomes of beef production systems, both recognized and implicit, are included in the experimental model. Only within this biological context is it possible for experimental techniques, such as PCA that was employed within this investigation, to be used to identify correlations between gene expression, disease, and both known and implicit sources of variation. An additional relevant limitation of this study is that it is underpowered for its overarching goal of identifying genes whose differential expression can be universally applied to all at arrival beef cattle to predict BRD within 28 days. However, our approach is an early, foundational event in support of this goal. Our demonstrated ability from ROC curves analysis, to predict calves at arrival that will develop BRD within 28 days, provides proof of concept for the eventual validation of predictive at-arrival biomarkers for clinical BRD. These findings also highlight the necessity to expand our approach to include cattle populations that will assure the appropriate power (i.e., number of animals and of unique herds) to characterize gene expression that predicts developing BRD in the face of gene expression variations that are characteristic of these populations.

Several studies have shown that production and economic loss increases with an increase in frequency of treatment and earlier timing of initial BRD treatment^11,57–59^. To align to this insight, and predict differences in future BRD severity, we stratified the severity of the BRD phenotype in our experiment. Cattle were separated into BRD severity cohorts based upon frequency of antimicrobial treatment, clinical assessment scores, and BRD-associated mortality. We identified 132 unique DEGs between the three disease cohorts. The increased DEGs identified in treated_1 cattle, when compared to both healthy and treated_2+, were largely involved with three major innate functions: neutrophil recruitment and degranulation, antimicrobial peptide production, and cellular cornification/keratinization. Increases in neutrophil recruitment/degranulation and antimicrobial peptide production traced primarily to increased differential expression of *CATHL1/2/3, CAMP, DSP, PRG3, AZU1, CTSG, CD177, MPO, LTF,* and *NGP*. These products are important for host antimicrobial defense, particularly involving leukocytic interactions within the airways^60–63^, and several of these gene products have been directly identified in cattle that were experimentally infected with BRD agents^13,14,60^.

Cattle produce all known mammalian classes of cathelicidins, compared to humans which only produce one, and research has primarily focused with their effects on bacterial pathogens^64–66^. Antimicrobial peptides, particularly cathelicidins, as well as *CD177*-mediated neutrophilic response in cattle, have been shown to be effective at mitigating Gram-negative bacterial infections and may be associated with modulating the cytotoxic/apoptotic host responses through the decrease of extracellular IL1A availability^61,64,67^. Dysregulation of these cytotoxic/apoptotic pathways is linked with poor clinical outcomes^60,68,69^. The increased cellular cornification/keratinization functions in treated_1 cattle versus healthy and treated_2+, identified through GO and pathway enrichment analyses, were impacted by increased expression of *KRT5/10/14/25, DSG1, DSP, CDSN,* and *SPINK5* in this group. While keratinization is primarily considered a modification of epidermal skin cells, this process has been shown to be an anti-apoptotic process of host barrier defense and structural repair in the lower airways related to infectious respiratory disease^70,71^.

To reduce dimensionality of the data and enable correlations between known sources of variability in our experiment and variability in gene expression to be identified, we performed principal component analysis that included both gene expression data and metadata for known variables for each animal. Component loadings identified positive correlation to disease severity and negative correlation to average daily gain in PC1, which were of moderate effect and statistically significant (Fig. 3B; FDR < 0.05). Accordingly, PC1, which by definition captures the greatest variability in the data, primarily measures the degree of weight loss and disease severity in BRD-affected cattle. This aligns to the known correlation between weight loss over time and disease in beef cattle^57,59^. In addition, *CFB*, a gene with the greatest positive influence on PC1 variation and a positive correlation to measures of disease severity using PCA, was also identified as acceptable discriminator of cattle that developed more severe disease (severe_2+) from cattle that remained healthy, based upon ROC analysis (Fig. 4; AUC = 0.769).

*CFB,* which was increased in both treated_1 and treated_2+ cattle when compared to healthy (Supplemental Table S4), encodes for complement factor B, the major acute phase protein needed for activation of the alternative pathway of complement. *CFB* is a pro-inflammatory molecule, produced by type II alveolar epithelial cells^72,73^. CFB can directly stimulate monocyte and B lymphocyte response to viral and bacterial respiratory pathogens^72–74^. *CFB* has been consistently identified as significantly expressed in BRD-afflicted cattle, both from pathogen challenge models and at-arrival sampling in naturally occurring BRD^13–18^. *CFB* is secreted by and activates M1 macrophages^75–77^. Our network analysis (Fig. 5; blue cluster) and IPA (Tables 1, 2) indicates that treated_2+ cattle, compared to treated_1 cattle, possess increased pro-inflammatory activity, which is driven by *IL1A* and *FADD*. This is made evident by the increased expression in macrophage-specific receptors *MARCO* and *CD163*, and decrease in the inhibitor glycoprotein *CD200R1*, when compared to both healthy and treated_1 cattle. Evidence suggests that *MARCO*, *CD163*, and *CD200R1* expression, induced by ligand binding to these receptors, causes a pro-inflammatory response by tissue macrophages, particularly in the lung, and may lead to an enhanced IL-1/IL-6 response^78–80^.

Through our previous and current research, we identified the differential expression of several genes involved in lipid transport and anti-inflammatory modalities^16^. Several genes that correspond to multidrug resistance-associated protein 4 (*ABCC4*/MRP4) were significantly decreased in treated_2+ cattle compared to both healthy and treated_1. MRP4 is a transporter protein found in multiple cell types and is directly involved in lipid molecule transport, such as prostaglandins and eicosanoids^81,82^. When prostaglandin E2 (PGE2) is produced via cyclooxygenase-2 (COX-2), MRP4 exports PGE2 to the extracellular space for pro-inflammatory signaling^81,83,84^. However, MRP4 also mediates the efflux of eicosanoids and its expression is coupled with an increase in *ALOX15* expression. *ALOX15* encodes for the lipid peroxidizing enzyme arachidonate 15-lipoxygenase, expressed by airway epithelium, circulating reticulocytes, macrophages, mast cells, and eosinophils^85–87^. *ALOX15* is required for the biosynthesis of specialized proresolving mediators (SPMs), such as resolvins and lipoxins^86,88^. These SPMs are important anti-inflammatory mediators derived from arachidonic acid and polyunsaturated fatty acids, and are responsible for metabolizing and suppressing the effects of prolonged inflammatory mediator signaling, including PGE2^81,86,88^. *ALOX15* production is implicated as a mitigating factor in a wide variety of inflammatory diseases in humans^89,90^. Regarding BRD, *ALOX15* has been found to be differentially expressed in animals challenged with single pathogens; importantly, those animals were all determined to exhibit mild clinical disease at time of sampling^13–15^. Additionally, at-arrival blood transcriptomes from this and previous research have shown cattle that remain healthy exhibit increased expression of *ALOX15*^16^. Thus, it can be hypothesized that cattle in these production settings are undergoing some form of cellular stress and subsequent pro-inflammatory signaling, but *ALOX15* upregulation and SPM production may be protective against severe clinical BRD.

We selectively evaluated the performance of six candidate mRNA biomarkers and their ability to predictively classify severity-based cohorts with ROC curves and calculated AUCs (Fig. 4). The genes evaluated in this analysis were identified as differentially expressed in both this current and our previous studies (*MARCO, CFB, MCF2L, ALOX15, LOC100335828* (*CD200R1*)*,* and *SLC18A2*)^16^. Moreover, five of these six genes were identified as differentially expressed when factoring for Year, Sex, and FEC status, with *LOC100335828* being the only gene considered non-significant in DESeq2 analysis (FDR = 0.1487). This approach demonstrated good-to-excellent discernment, at arrival and prior to the onset of clinical signs, of cattle that would develop severe BRD (treated_2+) within 28 days of arrival. Accordingly, these genes have inherent prognostic value for accurately identifying animals that require the highest frequency of treatment, yield the lowest weight gains overtime, and/or succumb to BRD-associated mortality. The ability to accurately predict cattle that are most likely to develop severe BRD could allow for precise management and antimicrobial treatment protocols, reducing the need to medicate beef cattle that are less likely to develop BRD during their production phases. Furthermore, predicting cattle that require multiple antimicrobial treatments, or eventually succumb to BRD, has significant economic impact. Several studies have established that beef cattle that receive multiple BRD treatments over their production phases yield lower carcass grades and weights, increased management and treatment cost per day, and overall reduced economic returns overtime^91–93^. Future research is necessary for evaluating these genes as prognostic indicators in larger and more complex beef cattle populations.

## Conclusion

This study was conducted to identify and/or corroborate at-arrival genes and genomic mechanisms that predict BRD and its severity. First, we expanded our prior analysis of gene expression in individuals from one beef production system to include individuals from two different beef production systems. Comparison of transcriptomes from these two populations with those of our previous and other researchers’ findings support a paradigm in which components of alternative complement, M1 macrophage and neutrophil activity, and lipid-mediated anti-inflammatory mechanisms influence clinical BRD outcomes. Cattle which were treated once and recovered from BRD had increased neutrophil recruitment and activation, antimicrobial peptide production, and cellular cornification/keratinization, compared to healthy cattle and treated_2+ cattle. Compared to healthy cattle, cattle that required two or more antimicrobial treatments and/or were euthanized due to BRD had increased expression of genes associated with pro-inflammatory modulation and response to cellular stress. Six genes identified in this study were previously identified as differentially expressed in at-arrival whole blood transcriptomes of cattle that developed BRD, and are considered candidate biomarkers for predicting BRD acquisition and severity: *MARCO, CFB, MCF2L, ALOX15, LOC100335828* (*CD200R1*)*,* and *SLC18A2*. Collectively, these findings may indicate that cattle treated only once for BRD are responding to natural BRD challenges more appropriately compared to cattle requiring multiple antimicrobial treatments. These findings show promise for predicting future clinical BRD cases in calves that lack overt signs of disease, as early as facility arrival. This would be anticipated to improve BRD morbidity and mortality and decrease antimicrobial use via earlier and more targeted antimicrobial administration. Attaining these outcomes necessitates investigations that improve understanding of the functional associations of these genes, and their validity as BRD biomarkers in larger, more complex cattle populations.

## Supplementary Information

Supplementary Figure S1.

Supplementary Figure S2.

Supplementary Tables.

## Data Availability

The sequencing data produced in this study were deposited to the National Center for Biotechnology Information Gene Expression Omnibus (NCBI-GEO) under the accession number GSE161396.
