# Supplementary material for: Multipopulational transcriptome analysis of post-weaned beef cattle at arrival further validates candidate biomarkers for predicting clinical bovine respiratory disease
Source: Sci Rep. 2021 Dec 13;11:23877. doi: 10.1038/s41598-021-03355-z (PMC8669006; doi:10.1038/s41598-021-03355-z)

**Multipopulational transcriptome analysis of post-weaned beef cattle at arrival further validates candidate biomarkers for predicting clinical bovine respiratory disease**

***Matthew Scott, Amelia Woolums, Cyprianna Swiderski, Andy Perkins, Bindu Nanduri, David Smith, Brandi Karisch, William Epperson, John Blanton**

*Correspondence:

Matthew Scott

[matthewscott@tamu.edu](mailto:matthewscott@tamu.edu); ORCID ID: 0000-0001-5243-7181

**Supplementary Figure S1**: A total of 209.4 million counts from 16,346 annotated genes resulted in a median library size of 43.6 million counts per sample. The x-axis represents each sample colored by their respective treatment cohort (blue: Healthy; red: Treated_1; yellow: Treated_2+). The y-axis represents the library size (number of gene counts). The black horizontal line represents the median library count across all 48 samples (43.6 million).


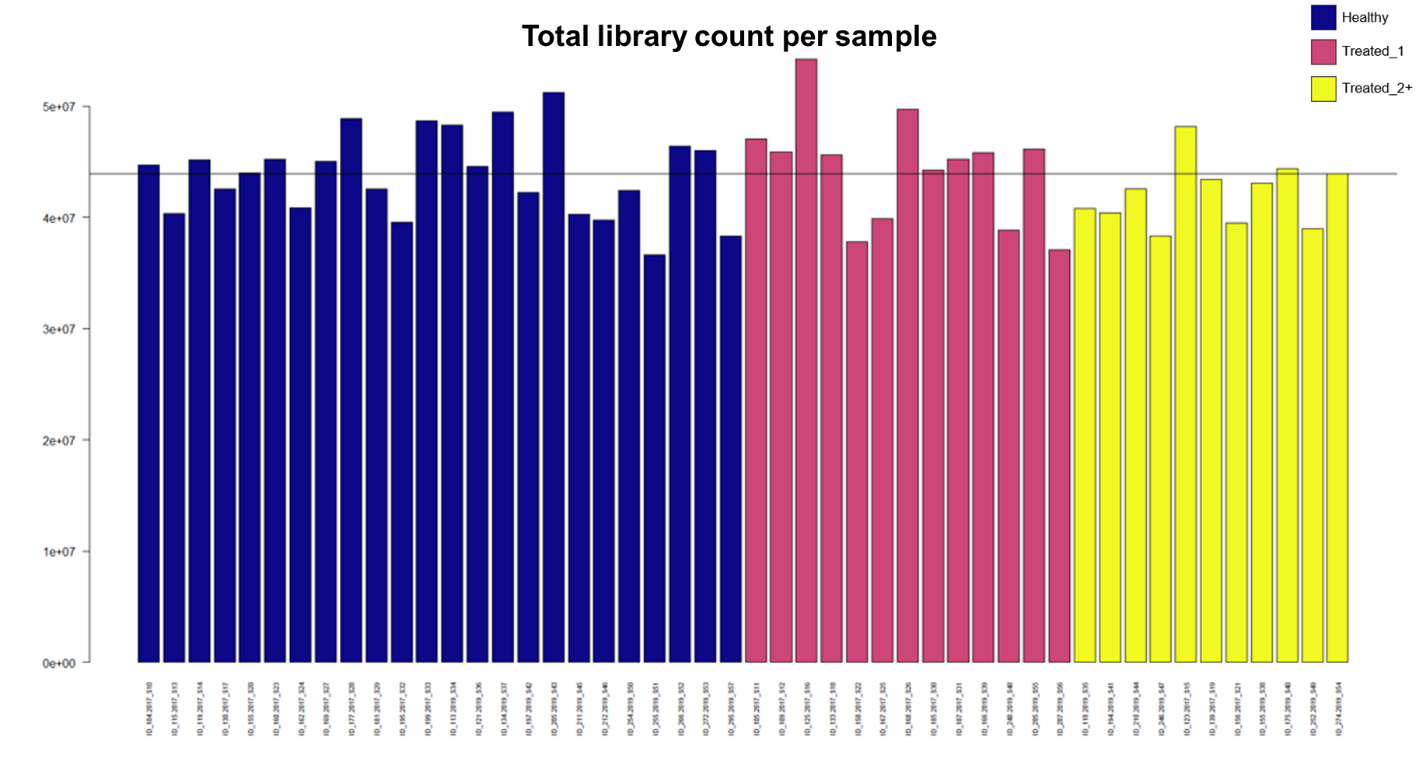

Supplement: Supplementary file 1 — Supplementary Figure S1. [file 41598_2021_3355_MOESM1_ESM.docx]
