# Supplementary material for: Multipopulational transcriptome analysis of post-weaned beef cattle at arrival further validates candidate biomarkers for predicting clinical bovine respiratory disease
Source: Sci Rep. 2021 Dec 13;11:23877. doi: 10.1038/s41598-021-03355-z (PMC8669006; doi:10.1038/s41598-021-03355-z)

**Multipopulational transcriptome analysis of post-weaned beef cattle at arrival further validates candidate biomarkers for predicting clinical bovine respiratory disease**

***Matthew Scott, Amelia Woolums, Cyprianna Swiderski, Andy Perkins, Bindu Nanduri, David Smith, Brandi Karisch, William Epperson, John Blanton**

*Correspondence:

Matthew Scott

[matthewscott@tamu.edu](mailto:matthewscott@tamu.edu); ORCID ID: 0000-0001-5243-7181

**Supplementary Figure S2**: K-means clustering of total gene expression identified twelve distinct clusters. The most severely diseased cattle (demarked by yellow (Treated_2+) and red (Dead) squares in the BRD_Severity row) tended to cluster to the right side of the heatmap. Heatmapping of these clusters was performed with scaled z-scores calculated from Trimmed Mean of M-values (TMM) normalized counts. Samples were labeled with population (year) and severity (including mortality) to illustrate differences in expression patterns. Yellow/white: relative high expression; purple/black: relative low expression.


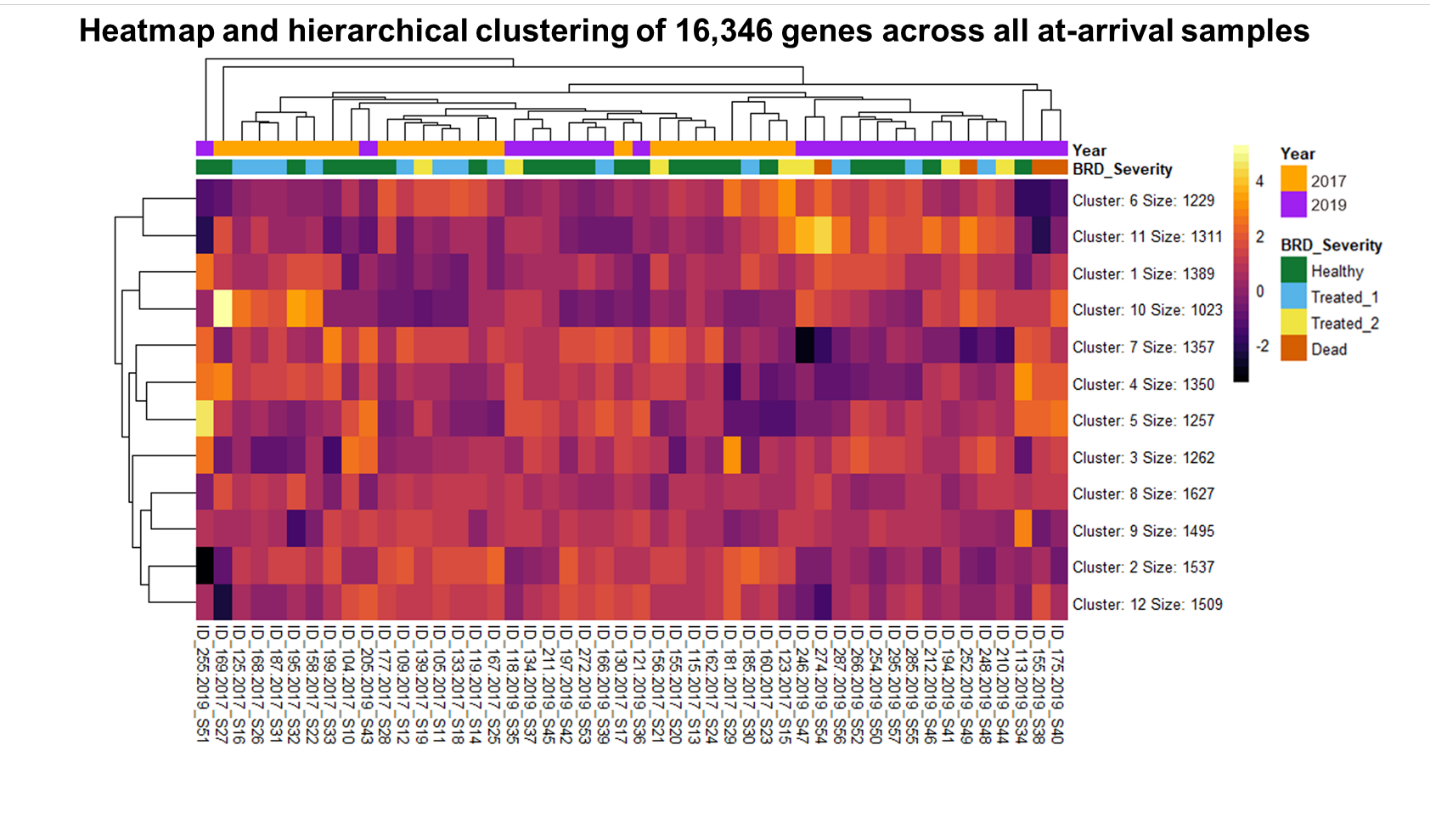

Supplement: Supplementary file 2 — Supplementary Figure S2. [file 41598_2021_3355_MOESM2_ESM.docx]
